# Influence of Reproductive Status on Occupancy of Salvage‐Logged Boreal Forest by Moose ( *Alces americanus* )

**DOI:** 10.1002/ece3.71302

**Published:** 2025-04-18

**Authors:** Julie P. Thomas, Mary L. Reid, Robert M. R. Barclay, Thomas S. Jung

**Affiliations:** ^1^ Department of Biological Sciences University of Calgary Calgary Alberta Canada; ^2^ Department of Environment Government of Yukon Whitehorse Yukon Canada; ^3^ Department of Renewable Resources University of Alberta Edmonton Alberta Canada

**Keywords:** camera traps, forest management, habitat use, occupancy models, trade‐off, ungulate

## Abstract

Wildlife‐habitat relationships reflect the behavioral choices made by species in response to perceived risks and rewards. Ungulates must often choose between habitats that provide forage and those offering concealment from predators, yet natural and anthropogenic disturbances create risky landscapes where tradeoffs may be difficult to navigate. Ungulate responses to forest disturbance may vary by sex and reproductive state, given that reproductive females with calves often prioritize predator avoidance. We investigated state‐dependent habitat use by reproductive and solitary moose ( *Alces americanus* ) in response to salvage logging after a widespread infestation by spruce beetle ( *Dendroctonus rufipennis* ) in the boreal forest of Yukon, Canada. We used camera traps and multistate occupancy models to examine moose occurrence in unsalvaged and salvage‐logged forests at different regenerative stages (0–10 years and 11–25 years postlogging) and levels of tree retention after logging. We compared results to single‐state occupancy models that did not account for reproductive status. As predicted, single‐state models showed high use of stands with low canopy cover and maximum tree removal (i.e., clear‐cuts). This suggested that moose capitalized on shrubby forage available in logged stands, regardless of regenerative stage. However, this result was overly simplistic. Multistate occupancy models revealed that forest age was the most important factor for female moose with calves, in contrast to solitary moose. Females with calves tended to avoid newly logged areas and preferred regenerating and unsalvaged forests with hiding cover, although estimates of effect size had low precision. Climate change is contributing to the rising frequency and severity of bark beetle outbreaks, and post‐infestation salvage logging has been implicated in the decline of moose populations in western Canada. Our results support the need to maintain diverse, mixed‐age forest landscapes to meet the food and cover requirements of moose in different demographic classes.

## Graphical abstract

We investigated state‐dependent habitat use by reproductive and solitary moose in response to salvage logging after a widespread spruce bark beetle infestation in Yukon, Canada. We used camera traps and multistate occupancy models to examine moose occurrence in unsalvaged and salvage‐logged forest stands at different regenerative stages (0–10 years and 11–25 years postlogging) and levels of tree retention. Female moose with calves tended to minimize predation risk by using protective forest cover and avoiding recently logged stands, in contrast to the preferences of nonreproductive moose.

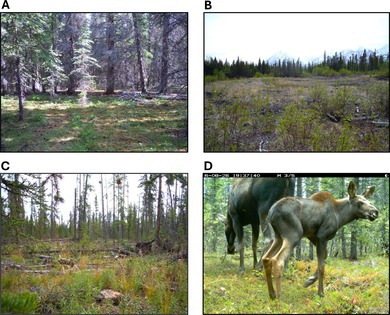

## 1 Introduction

For herbivorous mammals, habitat use involves trade‐offs between forage and cover from predators (Verdolin 2006). Demographic traits such as sex, age, and reproductive status may influence these trade‐offs (Bjørneraas et al. 2012; Fortin et al. 2022) and affect conservation outcomes, given the vulnerability of certain life stages (Wisdom et al. 2000). However, these demographic traits can be overlooked, particularly when species occurrence data are used for inference (e.g., citizen science observations or wildlife camera data; Crum et al. 2017; McKay and Finnegan 2023). In the case of sexually segregated ungulates, males and females may have different nutritional requirements and vulnerabilities to predators, causing sexually divergent habitat preferences (Ruckstuhl and Neuhaus 2002). Females with dependent young may choose habitats that minimize predation risk over those that maximize forage quantity or quality to increase calf survival (Marchand et al. 2015; Joly et al. 2016). Thus, females may avoid anthropogenic landscape features with reduced cover and increased predation risk, such as roads or forestry cut blocks (Dussault et al. 2012; Leblond et al. 2016; Mumma et al. 2019). This is particularly apparent during spring and summer, when calves are most vulnerable to predation (Francis et al. 2021). In contrast, male or solitary female ungulates may be relatively tolerant of human disturbance, taking greater risks in pursuit of forage (Grignolio et al. 2007). Ungulate population dynamics may be largely driven by calf survival and recruitment (Gaillard et al. 1998; Anderson et al. 2023). Thus, knowledge of female and calf responses to landscape disturbance can inform land use decisions that seek to protect important ungulate habitats.

Moose ( *Alces americanus* ) populations are declining across much of western North America (Kuzyk et al. 2018a; Wolf et al. 2021; Anderson et al. 2023). In western Canada, competing hypotheses about the proximate cause include elevated predation risk, hunting pressure, disease, and nutritional deficiencies (Anderson et al. 2023), but a leading hypothesis for the ultimate cause is landscape change (Kuzyk et al. 2018b). Unprecedented, large‐scale mountain pine beetle ( *Dendroctonus ponderosae* ) and spruce beetle ( *Dendroctonus rufipennis* ) outbreaks have occurred in British Columbia and the Yukon in recent decades, facilitated by climate change (Berg et al. 2006; Bentz et al. 2010). A surge of postinfestation salvage logging and road‐building activity may have created a risky landscape for moose (Kuzyk et al. 2018b; Boucher et al. 2022). Moose population declines are unlikely to be driven solely by adult survival, and low calf recruitment in response to salvage logging is a key hypothesis to explain declines (Anderson et al. 2023). Although early‐successional forestry cutblocks have abundant browse for moose (Gagné et al. 2016), they reduce concealment and escape cover until vegetation is sufficiently regenerated (Kunkel and Pletscher 2000; Hebblewhite et al. 2009). Early‐seral cutblocks increase food resources and attract grizzly bears ( *Ursus arctos* ) and black bears ( *Ursus americanus* ; Ciarniello et al. 2014; McKay and Finnegan 2022), both of which are significant predators of moose calves (Larsen et al. 1989; Ballard 1992; Wolf et al. 2021). Lastly, roads and trails associated with logging improve access and efficiency for wolves ( *Canis lupus* ; Whittington et al. 2011, Dickie et al. 2017) and hunters (Rempel et al. 1997), likely causing additive mortality and local population declines for moose (Boucher et al. 2022). Economic pressure to harvest beetle‐killed timber before it loses value (Corbett et al. 2016) can be a catalyst for permissive salvage logging practices such as large cutblocks, high annual allowable cut limits, and extensive road networks (Lindenmayer and Noss 2006; Patriquin et al. 2007). The cumulative pressures of natural disturbance and subsequent, intensive logging may have unpredictable and amplified effects on wildlife such as moose (Lindenmayer and Noss 2006).

Moose show variable responses to forest harvest, contingent on the scale of logging, stage of cutblock regeneration, silvicultural practices (e.g., tree retention, planting, and herbicide application), regional climate, and prior natural disturbance (reviewed by Johnson and Rea 2024). Sex and reproductive status introduce additional complexity. Occupancy‐based studies of moose‐forestry interactions often do not consider sex or reproductive status (Crum et al. 2017; Toews et al. 2018; Sand et al. 2021; McKay and Finnegan 2022). It is more common to consider these factors in radio telemetry studies, and evidence suggests that reproductive moose and solitary moose (i.e., males or nonreproductive females) select divergent habitat types to balance food‐predation trade‐offs (Oehlers et al. 2011; Joly et al. 2016; McCulley et al. 2017). However, state‐dependent responses to forestry are nuanced, and the literature is full of contradictions. Some research shows that males and nonreproductive females select cutblocks, which are relatively forage‐rich, whereas females with calves avoid them (Miquelle et al. 1992; Bjørneraas et al. 2011, 2012). Conversely, Mumma et al. (2021) observed female moose selecting regenerating cutblocks (9–24 years postharvest), regardless of their reproductive status. Female moose may sometimes prioritize forage over predation risk by selecting early‐seral cutblocks (Francis et al. 2021), despite increased mortality rates that may turn cutblocks into ecological traps (Boucher et al. 2022). Interactions between moose sex, reproductive status, and forestry may depend on additional contextual factors such as vegetation productivity (Gagné et al. 2016; Mumma et al. 2021), tree retention practices, and successional timelines (Johnson and Rea 2024). These complexities highlight the importance of expanding our knowledge on context‐ and state‐dependent moose responses to salvage logging, to avoid erroneous generalizations about moose habitat management.

Our objective was to evaluate habitat use by moose in response to salvage logging, following an outbreak of spruce beetle in the boreal forest of southwestern Yukon, Canada. We tested for different habitat preferences based on sex and reproductive status. Specifically, we examined the effects of different salvage logging practices on moose occupancy by comparing salvage‐logged stands of various tree retention levels and ages, and beetle‐affected stands that were not salvage‐logged. We sought to determine underlying mechanisms of moose response to logging in spring, summer, and fall (when calves are most susceptible to predation) by assessing stand‐ and landscape‐level characteristics that influence habitat use. We used motion‐triggered wildlife cameras and hierarchical, multistate occupancy models to assess the distribution of nonreproductive and reproductive females in response to salvage logging and associated roads. We contrasted these results with a single‐state occupancy model that did not account for sex or reproductive status. We hypothesized that (1) general moose occupancy (i.e., single‐state models) would indicate a preference for low‐retention logged stands with low cover and abundant forage, but females with calves (i.e., multistate models) would prefer high‐retention or unsalvaged stands with more tree cover; and (2) moose of any sex or reproductive status would prefer regenerating logged stands (11–25 years postharvest) over recently logged stands (0–10 years), owing to forage availability and greater lateral cover.

## 2 Materials and Methods

### 2.1 Study Area and Species

**FIGURE 1:**
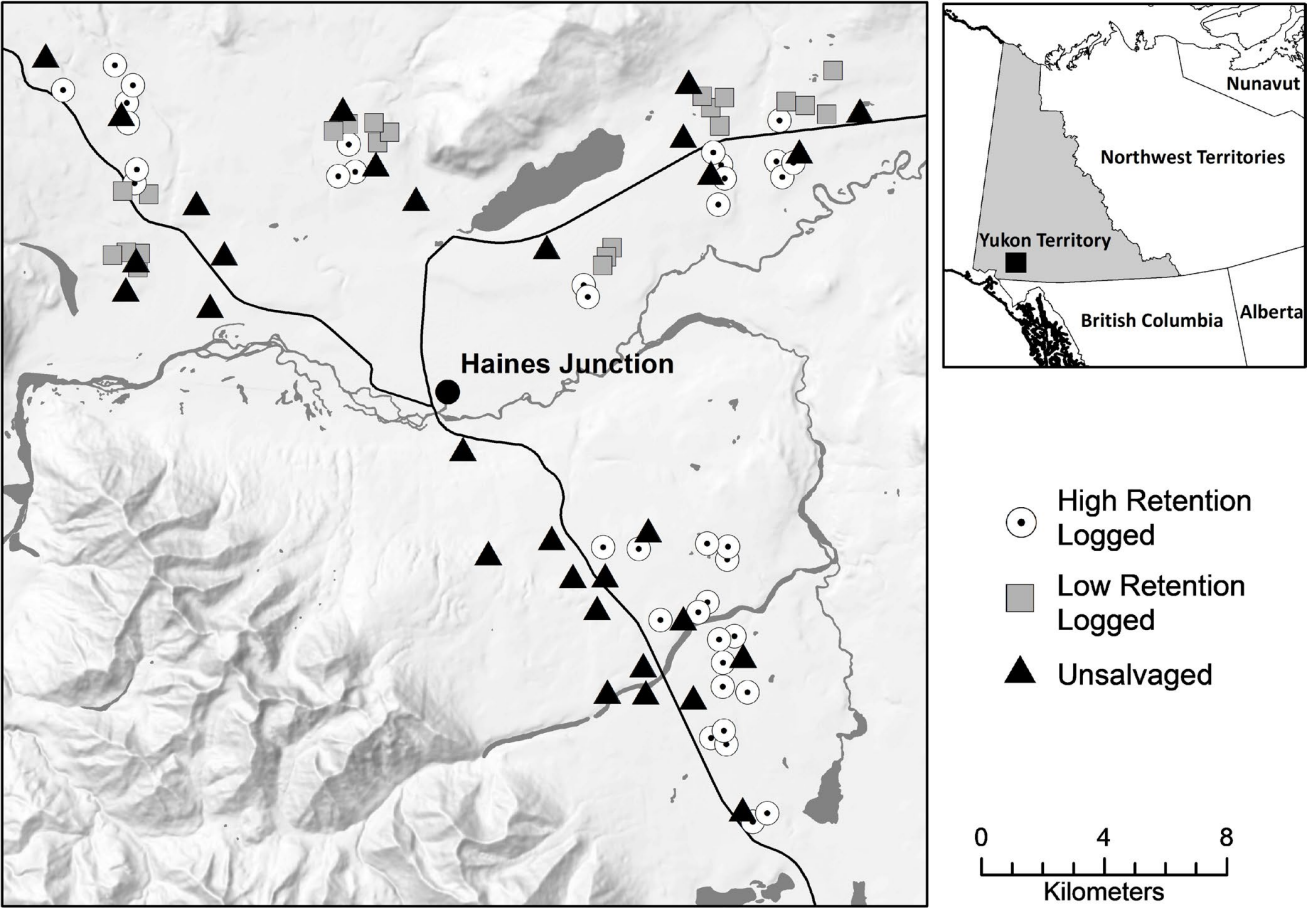
Study area and site locations in the boreal forest of southwestern Yukon, Canada. Wildlife cameras were placed in high‐retention logged (*n* = 38), low‐retention logged (*n* = 22), and unsalvaged spruce beetle stands (*n* = 30). Figure reprinted from Thomas et al. 2019 with permission from Elsevier.

Our study occurred in the boreal forest of southwestern Yukon, Canada (Figure 1). White spruce ( *Picea glauca* ) was the dominant overstory species, with willow (*Salix* spp.) understories and moss‐dominant groundcover. Trembling aspen ( *Populus tremuloides* ) stands were uncommon but found in areas that were previously logged or burned. A severe infestation of spruce beetles affected over 360,000 ha of forest in this region from 1990 to 2005 (Berg et al. 2006; Garbutt et al. 2006), killing approximately a third of all trees in affected stands (Randall et al. 2011). Very few mature spruce stands were not infested by spruce beetles in our study area. Ongoing salvage logging was initiated in the 1990's. In designated High Wildlife Value Areas, which cover more than 86% of the region, a 25% tree retention rate was recommended (Resource Assessment Technical Working Group 2006). However, clear‐cut logging was prioritized in areas where fuel reduction was crucial. Logging was generally small scale, with harvest blocks < 30 ha. Beyond postinfestation logging and the accompanying access roads, the landscape experienced minimal human disturbance.

Moose abundance in the study area declined by 44% from 1998 to 2008 (Westover et al. 2008). Wolves are important predators of moose in the Yukon (Hayes and Harestad 2000), but bears are the main predator of moose calves in our region (Larsen et al. 1989). Predation by grizzly bears is a potential limiting factor for moose populations in southwestern Yukon (Larsen et al. 1989). At the time of our study, moose hunting in the study area was closed to licensed hunters due to low moose abundance. However, a small amount of subsistence hunting by First Nations likely occurred.

### 2.2 Study Design and Surveys

**FIGURE 2:**
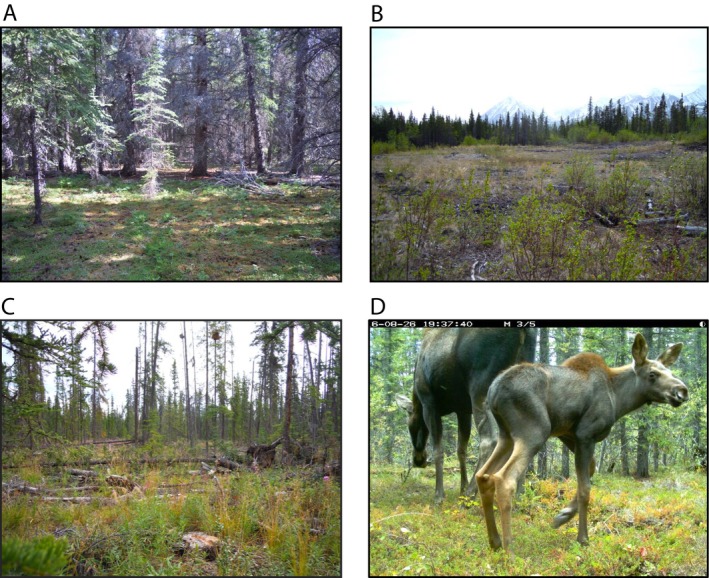
Photos of (A) an unsalvaged forest stand affected by spruce beetles; (B) a low‐retention salvage‐logged stand; (C) a high‐retention salvage‐logged stand; and (D) a female moose ( *Alces americanus* ) with a calf captured by a wildlife camera in southwestern Yukon.

We surveyed moose presence/absence, sex, age class, and reproductive status at 90 sites affected by spruce beetles, including 60 salvage‐logged stands and 30 unsalvaged stands (Figure 1). We surveyed salvage‐logged stands with different tree retention levels and age classes. We classified salvaged stands as high retention if postharvest overstory tree densities were 250–820 trees/ha (*n* = 38), and low retention if densities were 20–249 trees/ha (*n* = 22; Figure 2). Unsalvaged stands were also affected by spruce beetles, representing the reference condition for salvage‐logged stands. Unlogged stands had overstory tree densities of 818–4177 trees/ha (mean = 1780 trees/ha). Within the high retention class, 80% of the stands were newly logged (0–10 years old) and 20% were regenerating (11–25 years old). Within the low retention class, 37% were newly logged and 63% were regenerating. These classes represent significant stages of vegetation change relevant to moose in the boreal forest; newly logged stands have abundant forage, but little cover until they reach the regenerating stage (Fisher and Wilkinson 2005). We randomly selected unsalvaged sites from areas within 1 km of roads. Sampling sites were separated by ≥ 300 m, with an average of 741 m ± 484 SD between neighboring sites. Thus, we examined fine‐scale habitat use by moose rather than true patch occupancy, because moose home range sizes were much larger than spacing between cameras (Mackenzie et al. 2006; Efford and Dawson 2012).

We surveyed moose occurrence from 22 May to 12 October 2016 using 50 wildlife cameras (Hyperfire PC800, Reconyx, Holmen, Wisconsin, USA). Research in our study area indicated that data from camera traps were correlated with moose abundance obtained from traditional aerial surveys (Kenney et al. 2024). We rotated cameras through 90 sites in two phases; half were surveyed before 27 July and the rest afterward. Rotating cameras to a second site halfway through our study period allowed us to increase the number of sites sampled to improve the strength of inference. Sites were randomly assigned to the first and second survey period to achieve balanced representation of logged and unlogged sites across periods. The vast majority of moose calves are born within a two‐week period in mid‐May. Our sampling period corresponded to the time when 80%–100% of predation occurs for moose calves and adults in our region (Larsen et al. 1989). This is when we expected females with calves to be most risk aversive. We randomly selected camera locations within a given stand, positioned cameras on trees 50 cm above ground, and aimed them at forest openings to increase wildlife detection rates. We used Timelapse 2 software (Greenberg Consulting Inc., Calgary, Alberta, Canada) to classify wildlife images. Detection events of the same species were separated by a minimum of 30 min. Male moose were identified by the presence of antlers or pedicels, whereas females had vulva patches and no antlers or pedicels (Rea et al. 2013). Vulva patches and absence of antlers were ideal for confirming females, but both features were not always visible in photographs, so either could be used to distinguish females from males. Females with calves were considered reproductive, whereas those without calves were not. Calves were easily distinguished based on their diminutive body size during our sampling period. All photos were classified by the same individual and checked for accuracy by another.

### 2.3 Habitat Variables

**FIGURE A1:**
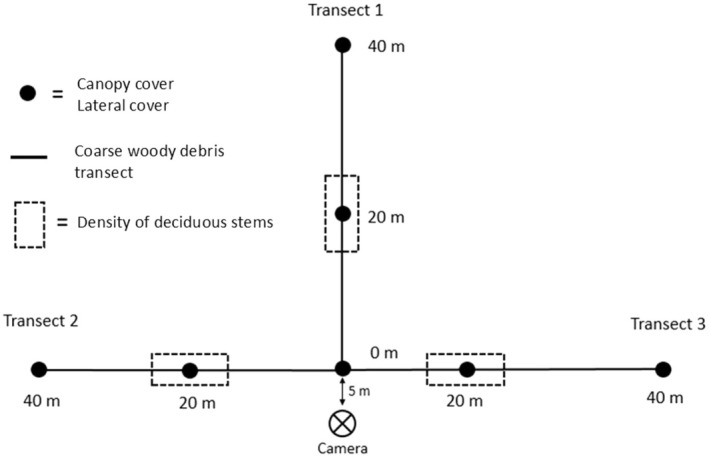
Diagram of transect and plot design for measuring site‐specific habitat variables around wildlife cameras. Variables included canopy cover (%), lateral cover (%), coarse woody debris (CWD; logs/120 m), and density of deciduous stems (stems/10 m^2^).

We measured site‐specific forest structure to characterize fine‐scale habitat value in terms of food and cover (Table 1). We recorded site vegetation characteristics along three 40‐m transects originating at the camera location and forming a “T” shape (Figure A1). At each of seven sampling points (central camera location and 20‐m and 40‐m markers on each transect), we recorded canopy cover (%) with a convex spherical densiometer. We measured lateral cover (%) at each point with a 2‐m tall cover pole, taking readings at 5‐m distances from the sampling point in each cardinal direction (Griffith and Youtie 1988). We counted the number of deciduous stems within three 1 × 10 m rectangular plots (stems/10 m^2^), including all shrubs and deciduous trees ≤ 2 m tall. We measured the abundance of coarse woody debris (hereafter CWD) by counting the number of downed trees (≥ 4 cm diameter) intersecting each of the 40‐m transects (Forester et al. 2007).

**TABLE 1:** Site‐ and landscape‐scale predictor variables, descriptions, and associated hypotheses and predictions for moose ( *Alces americanus* ) occupancy in salvage‐logged and unlogged forests in Yukon, Canada.

Predictor	Description	Hypothesis and prediction (+ or ─)
Stand Type	Low‐retention logged, high‐retention logged, unsalvaged	Logging increases forage (Gagné et al. 2016) but reduces cover (nonreproductive moose +; females with calves −) (Bjørneraas et al. 2011)
Stand Age	Early‐seral (0–10 years) and mid‐seral (11–25 years)	Cover and food availability increase over time as logged stands regenerate (+) (Mumma et al. 2021)
Canopy	Canopy cover (%) of stand calculated with a spherical crown densiometer	Canopy reduces understory productivity and food (nonreproductive moose −), but tree cover also reduces predation risk (females with calves +) (Bjørneraas et al. 2011, Gagné et al. 2016; Johnson and Rea 2024)
Deciduous Stems	Number of deciduous stems (stems/10 m^2^), including shrubs and trees < 2 m tall	Deciduous shrubs/trees are preferred forage species and increase food availability (+) (Spitzer et al. 2021; Koetke et al. 2023)
Lateral Cover	Percent cover (%), measured with a 2 m tall cover pole	Lateral cover increases visual obstruction and reduces predation risk (+) (Kunkel and Pletscher 2000; White and Berger 2001)
CWD	Total number of downed trees with diameter > 4 cm along three 40 m transects	Coarse woody debris (CWD) increases energetic cost of travel and impedes escape (−) (Kuijper et al. 2013)
Water Distance	Distance (km) to nearest wetland, lake, or river	Waterbodies provide forage (i.e., aquatic plants) (MacCracken et al. 1993; Tischler et al. 2019); moose occupancy will decline with increasing distance from water and riparian habitat (−)
Linear Density	Linear disturbance density (km/km^2^) within 500 m of camera	Improves access for predators and hunters, perceived as higher risk by moose (−) (Laurian et al. 2012; Mumma et al. 2021)
Forest Cover	Unsalvaged forest cover (%) within a 500 m radius of camera	Foraging habitat near forest cover provides a balance of food and nearby escape cover (+) (Courtois et al. 2002)
Bears	Camera detection rate (detections/day) of grizzly bears and black bears	Bears are major predators of moose calves (Ballard 1992); females avoid areas with high bear abundance (−)

*Note:* The predicted effects on moose occupancy are shown in parentheses (+ or −).

To characterize moose habitat at the landscape scale, we generated spatial land cover data through classification of Sentinel‐2 satellite images from the European Space Agency Copernicus Program (existing landcover data were not available). We used supervised maximum‐likelihood classification in ArcGIS version 10.4.1 (ESRI, Redlands, California, USA) to distinguish forests, water, and open habitat (e.g., cutblocks and meadows). Classification accuracy was 90%, verified with ground truthing and aerial imagery at 100 locations. We calculated forest cover (%) within a 500‐m radius of each site using Fragstats version 4.2.1 (University of Massachusetts, Amherst, Massachusetts, USA). We measured the distance to the nearest permanent water body (lakes, wetlands, and streams) and the nearest forest edge (i.e., logged stand or linear feature) with the Point Distance tool. However, forest edge was omitted from all models due to strong collinearity with other variables. Lastly, we calculated the density of linear disturbances within 500 m (km/km^2^; roads, transmission lines, pipelines, and cutlines) using the Line Density tool in ArcGIS. Elevation and aspect often have a strong influence on moose habitat use in mountainous regions (e.g., McCulley et al. 2017). However, our study was in a relatively flat valley with little topographic variation (Figure 1), and we omitted both variables.

While both grizzly bears and black bears are key predators of moose calves in our region (Larsen et al. 1989), the relative risk that each species posed to moose calves in our specific system was unknown. As such, we used the combined camera detection rate of grizzly bears and black bears (bear detections/month) as a proxy for predation risk for moose calves (Darlington et al. 2022). Wolf densities were unknown in the study area, but wolves were only detected at two camera sites. Thus, we did not consider wolf presence as a proxy for predation risk.

### 2.4 Statistical Analyses

We applied occupancy models to camera data using the RPresence package in R (R Core Team 2017). This package is an R interface for running occupancy models in Program Presence (v. 2.13.42; Hines 2006). This method estimates species occurrence in relation to environmental variables, accounting for imperfect detection (Mackenzie et al. 2006). In the context of occupancy models, detection probability refers to the likelihood of detecting a species, given that a site is occupied. Detection probability is calculated from repeated surveys. The model assesses how site characteristics influence species occupancy (*ψ*) and how survey conditions affect detection probability (*ρ*).

First, we developed a single‐state model for general moose occupancy (i.e., moose of any sex or reproductive status) as a baseline for comparison. We then created multistate occupancy models to compare habitat use by solitary females and females with dependent calves (Nichols et al. 2007; Mackenzie et al. 2009). Models were parameterized as in Nichols et al. 2007, with three hierarchical occupancy states: unoccupied by female moose (state 0); occupied by female moose (state 1); and occupied by female moose with calves (state 2). These are captured by two parameter estimates: *ψ* ^1^ is the probability of occupancy by female moose and *ψ* ^2^ is the probability of occupancy by calves, given that a site is occupied by female moose. Male moose were not included in state 1, as this would violate the hierarchical nature of the models. Multistate models allow for state uncertainty and for detection probabilities to vary by state. For example, adult females may be easier to detect than their calves (Bergman et al. 2020). Detection probabilities were parameterized as follows: *ρ* ^1^ = probability that female moose are detected when true state = 1; *ρ* ^2^ = probability that female moose are detected when true state = 2; and *δ* = probability that calves are detected when present. We constrained *ρ* ^1^ to equal *ρ* ^2^ (hereafter referred to as *ρ*) because this parameterization improved model fit and parsimony. We assumed that reproductive state remained constant within a season (i.e., May–July and July–October) to meet the closure assumption of occupancy models (Kendall et al. 2009). It is unlikely new calves were born after early June, and calves were not weaned during these periods (Testa 2004), but some calf mortality likely occurred.

We divided continuous wildlife camera data into discrete sampling periods of 15 days, yielding minimum detection probabilities that were adequate (> 0.2) for models to converge on precise occupancy estimates (Mackenzie and Royle 2005). Before running occupancy models, we determined the most plausible detection probability model by fitting different combinations of site‐ and survey‐specific predictor variables. We then incorporated the best detection model into subsequent occupancy models. Dense vegetation may reduce the camera detection zone or influence animal behavior in a way that affects the probability of detection (Burton et al. 2015). We considered lateral cover and sampling month as predictors of general moose detection. Calves are more difficult to detect during the birthing season in May and in fall when calves are more independent and wander further from females (Bergman et al. 2020). By fall, calves are more likely to have suffered predation mortality during preceding months. Thus, we included state (solitary females versus females with calves) and month as detection predictors for multistate models. We followed a step‐wise process for developing occupancy models. We first found the most supported detection model, then evaluated the strongest predictors of occupancy (Gould et al. 2019). In the case of multistate models, we incorporated an additional step: we held the best *ψ* ^1^ (female occupancy) model constant while we evaluated predictors of *ψ* ^2^ (occupancy of females with calves).

We developed a set of models to test a priori hypotheses about the influence of salvage logging and forest structure on moose single‐state and multistate occupancy (Burnham and Anderson 2004). The candidate set of occupancy models included single‐variable models and additive combinations of variables representing food, cover, or predation risk (Table 1, Table A1). We standardized continuous predictor variables by converting to *z*‐scores. Correlated predictors (*r* > 0.6) were not included in the same models (Dormann et al. 2013).

We compared candidate models against null and global models using Akaike's Information Criterion (AIC). We excluded models with uninformative parameters (complex models outperformed by simpler nested models) and those failing to converge (Arnold 2010). We did not use AIC corrected for small sample size (AICc) due to potential bias for non‐Gaussian data, particularly logistic models (Richards 2015). We used quasi‐AIC (QAIC) values to compare models when overdispersion and a lack of fit were indicated by goodness‐of‐fit tests (i.e., $\hat{c}$ >1; MacKenzie and Bailey 2004). When QAIC was used for model selection, we inflated unconditional standard errors by the square root of the $\hat{c}$ value (MacKenzie and Bailey 2004). Relative model strength was assessed via AIC or QAIC weights. If no model had a weight > 90%, we averaged parameter estimates and unconditional standard errors across a set of confidence models (the “confidence set”) totaling approximately 95% Akaike weight (Burnham and Anderson 2004). We model‐averaged 95% confidence intervals via the delta method. Unconditional standard errors were transformed to the logit scale, then confidence intervals were calculated and back‐transformed to the probability scale, as implemented with the modAvg function in RPresence.

We assessed spatial autocorrelation in occupancy model residuals (Warton et al. 2017) from the top‐performing occupancy model (lowest AIC) using Moran's I correlograms (Dormann et al. 2007). Methods had not been developed for calculating residuals from multistate occupancy models at the time of this research (Warton et al. 2017), so we prepared correlograms from raw presence‐absence data for the analysis of females with calves (e.g., Webb et al. 2014). We evaluated the fit of the most saturated model via bootstrapping with 10,000 runs and a chi‐square goodness‐of‐fit test (MacKenzie and Bailey 2004).

## 3 Results

Camera surveys were successful at 89 of 90 sites (one camera malfunctioned at an unsalvaged site), resulting in 5905 camera‐trap days. Moose were detected at 49% of sites, with variable detection rates by sex and reproductive status (male moose: 28% of sites; female moose of either state: 36%; females with confirmed calves: 12%). Two of 104 independent moose detections could not be classified as male or female (1.9% of all moose detections); these detections were retained in the model for general moose occupancy but omitted from the state‐dependent model. Black bears and grizzly bears were detected at 19% and 7% of sites, respectively. Among sites with bears, detection rates varied from 0.38 to 3.9 detections/month. Bear detection rates were higher in logged stands (mean = 0.23 detections/month ±0.076 SE) than in unsalvaged stands (mean = 0.14 detections/month ±0.085).

**FIGURE A2:**
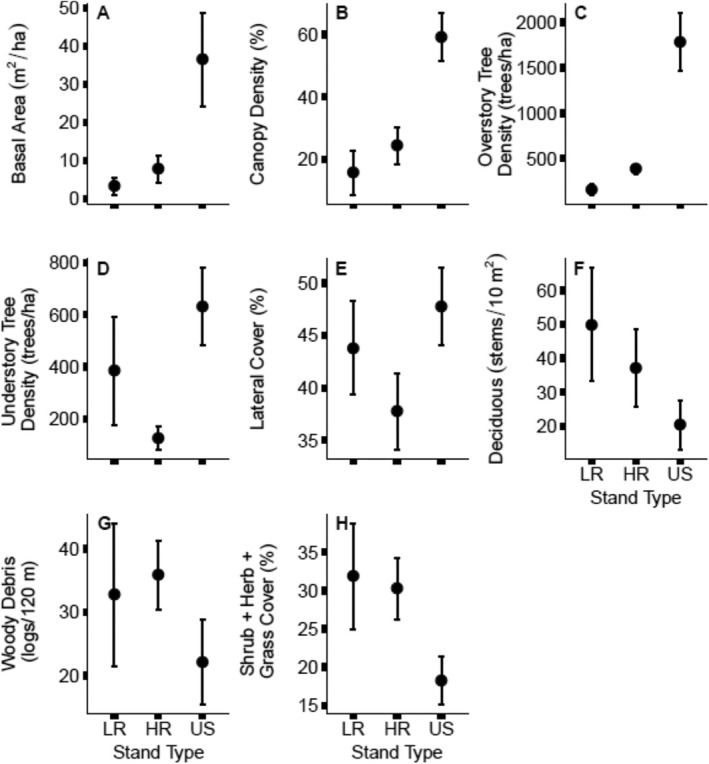
Comparison of mean vegetation and forest structure measurements between low‐retention salvage‐logged (LR), high‐retention salvage‐logged (HR), and unsalvaged beetle‐affected stands (US) in southern Yukon, Canada, including: Basal area (A), canopy cover (B), overstory tree density (C), understory tree density (D), lateral cover (E), deciduous stem density (F), course woody debris (G), and shrub + herb + grass cover (H). Error bars represent 95% confidence intervals.

**FIGURE A3:**
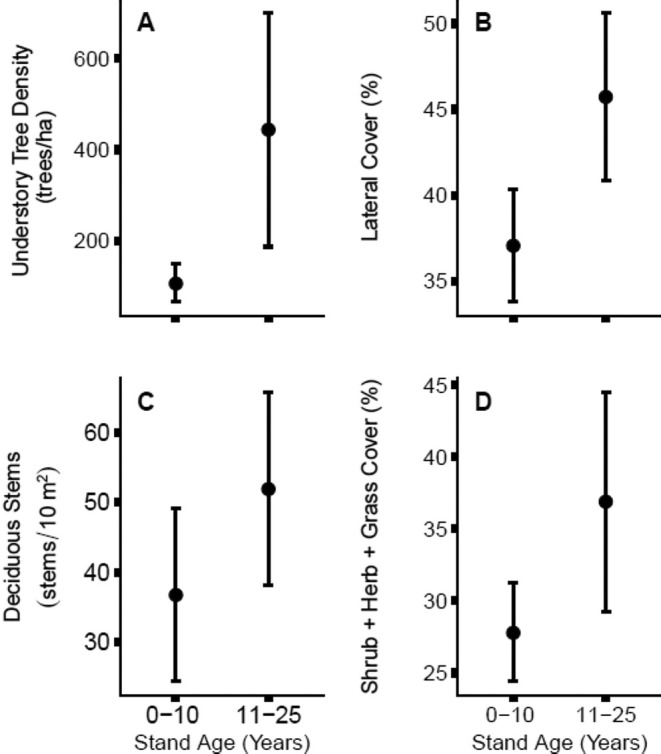
Comparison of mean vegetation and forest structure measurements between young salvage‐logged stands (0–10 years postharvest) and regenerating salvage‐logged stands (11–25 years postharvest) in southern Yukon, Canada, including: Understory tree density (A), lateral cover (B), deciduous stem density (C), and shrub + herb + grass cover (D). Error bars represent 95% confidence intervals.

Salvage‐logged stands were characterized by less canopy cover and lateral cover, higher deciduous stem density, and more CWD than unsalvaged stands. Regenerating logged stands (11–25 years) had more lateral cover and higher shrub densities than newly logged stands (0–10 years). Stand‐level vegetation characteristics by tree retention and age class are presented in the Appendix (Figures A2 and A3).

### 3.1 General Moose Occupancy (Single‐State Models)

Lateral cover and month did not have an influence on the probability of detection; the top‐ranked moose detection model was the null model. Model‐averaged detection probability for moose was 0.24 ± 0.04 and site occupancy varied from 0.18 to 0.98.

Seven models were included in the 95% confidence set predicting general moose occupancy (Table 2, Table A1). The top model (canopy cover + distance to water + CWD) had six times more support than the next best model in the candidate set, based on QAIC weights (QAIC weight of top model [0.66]/QAIC weight of alternative model [0.11]). Canopy cover, distance to water, and CWD each appeared in four of seven models in the confidence set, and stand type (high‐retention logged, low‐retention logged, unsalvaged) appeared in three models. The null model was not supported by the data (QAIC weight = 0.006, Table A2).

**TABLE 2:** Confidence set of occupancy (*ψ*) models for moose in beetle‐affected and salvage‐logged forest in Yukon, Canada.

Species/Model	QAIC	ΔQAIC	QAIC weight	K
Single‐State Moose Occupancy				
*ψ* (Canopy + CWD + Water Distance), *ρ* (.)	244.56	0	0.66	5
*ψ* (Canopy + CWD), *ρ* (.)	248.14	3.58	0.11	4
*ψ* (Stand Type + CWD + Water Distance), *ρ* (.)	249.46	4.9	0.06	5
*ψ* (Canopy + Water Distance), *ρ* (.)	249.73	5.17	0.05	4
*ψ* (Stand Type + CWD), *ρ* (.)	250.67	6.11	0.03	4
*ψ* (Canopy), *ρ* (.)	250.99	6.43	0.03	3
*ψ* (Water Distance + Stand Type), *ρ* (.)	251.15	6.59	0.02	4
Multistate Moose Occupancy				
*ψ* ^1^ (Canopy + CWD), *ψ* ^2^ (Stand Age), *ρ* (.), *δ* (.)	295.06	0	0.73	7
*ψ* ^1^ (Canopy + CWD), *ψ* ^2^ (Water Distance + Deciduous Stems), *ρ* (.), *δ* (.)	300.03	4.97	0.06	8
*ψ* ^1^ (Canopy + CWD), *ψ* ^2^ (Deciduous Stems), *ρ* (.), *δ* (.)	300.58	5.52	0.05	7
*ψ* ^1^ (Canopy + CWD), *ψ* ^2^ (Water Distance + Bears), *ρ* (.), *δ* (.)	300.62	5.56	0.04	8
*ψ* ^1^ (Canopy + CWD), *ψ* ^2^ (Water Distance), *ρ* (.), *δ* (.)	301.14	6.08	0.04	7
*ψ* ^1^ (Canopy + CWD), *ψ* ^2^ (Stand Type + Bears), *ρ* (.), *δ* (.)	302.43	7.36	0.02	8
*ψ* ^1^ (Canopy + CWD), *ψ* ^2^ (Bears), *ρ* (.), *δ* (.)	302.97	7.91	0.01	7

*Note:* Quasi‐Akaike's information criterion (QAIC), delta QAIC, QAIC weight, and number of parameters (*K*) are shown for each candidate model. Multistate models include two occupancy states: Probability of occupancy by adult females (*ψ* ^1^), and probability of occupancy by females with calves (*ψ* ^2^). *ρ* = probability of detection of adult females (true state = 1 or 2), and *δ* = probability that calves are detected, given occupancy (true state =2). Occupancy predictors are defined in Table 1 and (.) refers to the null model.

**FIGURE 3:**
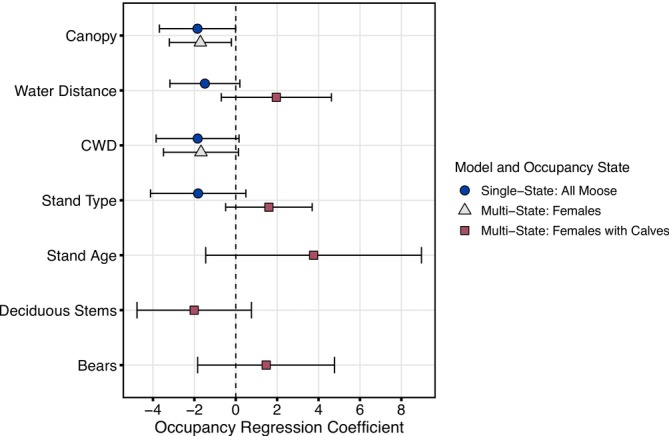
Model‐averaged regression coefficients (points) and 95% unconditional confidence intervals (error bars) for single‐state occupancy models (general moose occupancy, *ψ*) and hierarchical multistate occupancy models (occupancy by adult females, *ψ* ^1^, and females with calves, *ψ* ^2^). Estimates were calculated by averaging across the set of confidence models where QAIC or AIC weights summed to 95%. Occupancy predictors are defined in Table 1.

**FIGURE 4:**
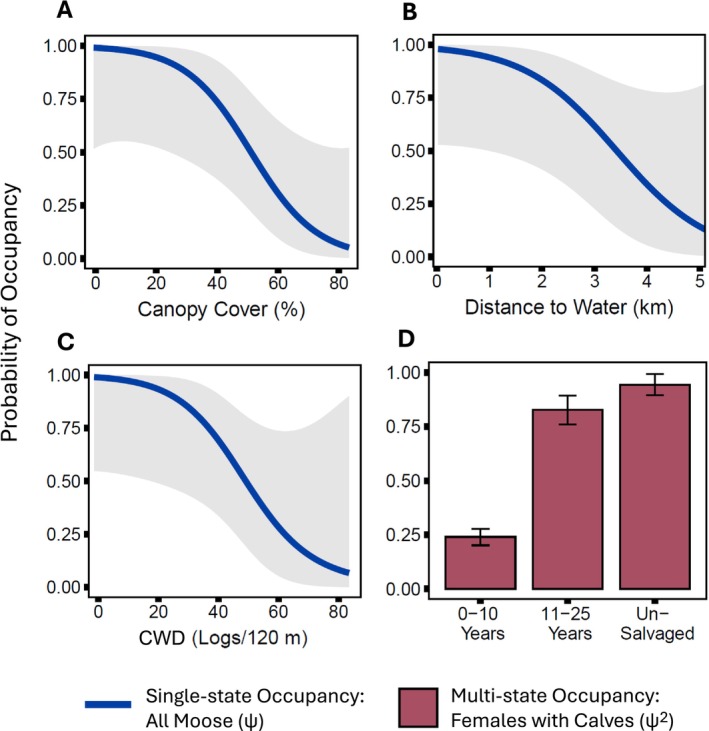
Model‐averaged estimates of moose ( *Alces americanus* ) occupancy as a function of canopy cover, distance to water, coarse woody debris (CWD), and stand age in southwestern Yukon, Canada. Results of single‐state occupancy models (general moose occupancy, *ψ*) are shown with blue lines, and multistate models (females with calves, *ψ*2) are shown with red bars. Occupancy estimates (bar plots) and predicted relationships (lines) were obtained by model‐averaging across the confidence set of models where QAIC weights summed to 95%. Lines show predicted effects when other predictor variables are held constant at their mean. Shaded regions and error bars are unconditional 95% confidence intervals.

Canopy cover had a statistically significant effect on single‐state (general) moose occupancy (i.e., 95% confidence intervals did not overlap zero; Figure 3, Table A2). General moose occupancy declined with increasing canopy cover (Figure 4A). General moose occupancy tended to decline with increasing distance to water (Figure 4B) and increasing CWD (Figure 4C), but confidence intervals overlapped zero, indicating either inconsistent effects or imprecise parameter estimates (Figure 3, Table A2). Mean model‐predicted occupancy values were generally highest in low‐retention logged stands (0.93 ± 0.03 SE), followed by high‐retention logged stands (0.76 ± 0.06) and unsalvaged stands (0.57 ± 0.08), but stand type was not a statistically significant predictor (Figure 3, Table A2). Stand age, deciduous stem density, lateral cover, forest cover, and linear disturbance density had no influence on general moose occupancy.

**FIGURE A4:**
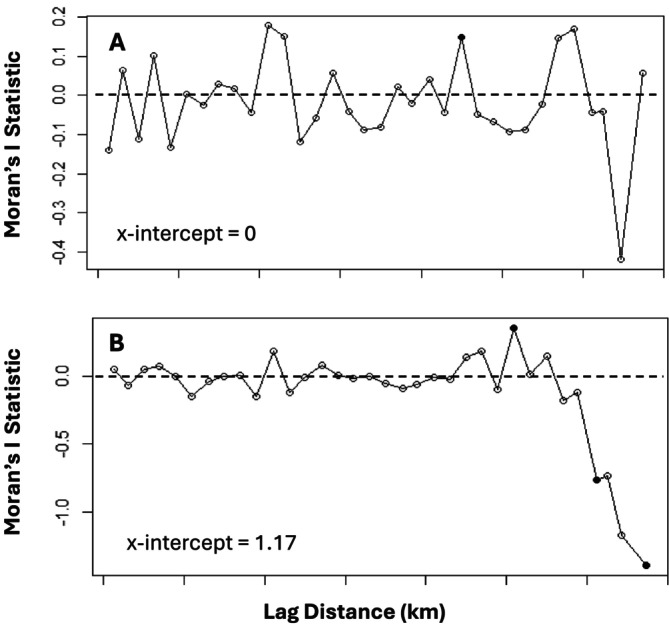
Moran's I spatial correlogram, showing the results of spatial autocorrelation (Moran's *I*) tests across consecutive 1 km distance classes (lag distance) on the residuals of occupancy models for (A) single‐state models, and (B) multi‐state models. Moran's *I* statistic values range from −1 to 1, and the *x*‐intercept represents the distance at which sites are no more similar than expected by chance. Closed circles represent significant spatial autocorrelation (i.e., *p* < 0.05). Dotted lines show zero correlation, for reference.

The goodness‐of‐fit test of the global model indicated no lack of fit (*p* = 0.11), but data were overdispersed ($\hat{c}$ = 1.3). No spatial autocorrelation was detected in model residuals; the *p*‐value for the Moran's *I* test was > 0.05 at lag distances up to the maximum of 35 km, and the xintercept of the correlogram was 0‐km (Figure A4).

### 3.2 Occupancy by Reproductive Status (Multistate Models)

Detection probability for female moose (*ρ*) was not significantly influenced by month or reproductive state, and calf detection (*δ*) was not influenced by month, as the null model was best for predicting these detection parameters. The model‐averaged estimate of *ρ* was 0.18 ± 0.04 and *δ* was 0.46 ± 0.14.

Following a step‐wise procedure for multistate occupancy, we first investigated the top predictors of occupancy by female moose (*ψ* ^1^). The best supported model for female moose included canopy cover and CWD (AIC weight = 0.87 in a set of 13 models), sharing two of the top three predictors of general moose occupancy. Consistent with single‐state models for general moose occupancy, canopy cover had a significant negative effect on occupancy by female moose (Figure 3, Table A3). Female moose occupancy generally declined with increasing CWD, but confidence intervals overlapped zero (Figure 3, Table A3). We held the female moose model constant (canopy cover + CWD) as we evaluated specific predictors of occupancy by females with calves (*ψ* ^2^).

Occupancy by female moose with calves was best predicted by stand age, which was not a predictor of occupancy by moose in general (single‐state models) (Table 2). The stand age model (AIC weight = 0.73) was 12 times more likely than the next best model for females with calves (Table 2, Table A1). The estimated effect of stand age was positive (*β* = 3.76 ± 2.67), although imprecise, with 95% confidence intervals overlapping zero (Figure 3). The model‐averaged prediction of occupancy by females with calves was 0.24 ± 0.04 in newly logged stands (0–10 years postharvest), which was significantly lower than in regenerating stands (0.83 ± 0.07; 11–25 years postharvest) and unsalvaged stands (0.94 ± 0.05; Figure 4D).

The null model was the lowest‐performing model with an AIC weight of 0.0003, indicating that predictor variables—particularly stand age—greatly improved the explanatory power of multistate models (Table A1). Strong spatial autocorrelation was not detected in raw presence/absence data (Moran's I statistic was < 0.35 at all lag distances and *p* > 0.05 for most lag distances; Figure A4). Aside from stand age, other models in the candidate set for females with calves included water distance, deciduous stems, bears, and stand type (Table 2, Table A1). However, model weights were low (≤ 0.06), and these predictors did not have a statistically significant effect on occupancy (Figure 3, Table A3).

## 4 Discussion

Reproductive status influenced moose occupancy in a salvage‐logged landscape. Our data indicated that females with calves tended to use different habitats than solitary moose. Limiting factors for moose populations, such as predation and nutrition, have disproportionate effects on calf survival and recruitment (Joly et al. 2017). Rates of adult moose survival are typically high (> 85%; Joly et al. 2017), but calf survival rates are low, particularly in declining populations (e.g., < 30%; Testa 2004; Wolf et al. 2021). Thus, to develop moose conservation strategies in managed forests, the logging response of females with calves is more informative than that of the overall moose population. Understanding habitat use by reproductive individuals provides a link between animal space use, fitness, and population dynamics (McLoughlin et al. 2006; Dussault et al. 2012).

Our results suggest a state‐dependent tradeoff between foraging and predation risk, the balance of which is tipped towards forage for the general moose population, but towards risk avoidance for females with calves. As predicted, results of models that did not account for moose sex or reproductive status (i.e., single‐state occupancy models) supported a significant preference for open stands with minimal tree cover. Moose in our region are shrub‐specialists, with > 80% of their summer diet comprised of shrubs (Jung et al. 2015). The attraction of logged stands could be attributed to abundant deciduous shrubs relative to surrounding low‐productivity spruce forest (Figure A2). Other boreal forest studies found that moose selected regenerating clearcuts with large quantities of forage (Rempel et al. 1997; Dussault et al. 2005; Anderson et al. 2018). However, silvicultural practices can reduce the nutritional quality of moose browse in cutblocks, even if forage is abundant (Johnson and Rea 2024). Moose in a heavily logged landscape in British Columbia had more diverse diets and ate fewer shrubs, perhaps because herbicide application and mechanical brushing reduced the nutritional quality of shrubs (Koetke et al. 2023). Forage quality may further decline as a result of low soil moisture and intense solar radiation in cutblocks (Happe et al. 1990). Logged stands in our study were regenerating naturally without the use of herbicides or brushing, which may have reduced any loss of forage quality.

In contrast to solitary moose, female moose with calves were more likely to use unsalvaged forests—particularly in comparison to newly logged stands (0–10 years postharvest)—although effect sizes were uncertain and confidence intervals overlapped zero. By selecting unlogged forest habitat with abundant cover, reproductive females can potentially reduce calf mortality (Severud et al. 2019). They may also decrease anti‐predator vigilance in forests with abundant cover, allowing more time to forage and meet the energetic demands of lactation and calf‐rearing (White and Berger 2001). However, forage‐risk tradeoffs are seasonally variable. For example, female moose in British Columbia selected low‐risk habitats during calving and fall seasons, but chose disturbed habitats with abundant forage during the rest of the year (Francis et al. 2021). In our study, tree retention levels (i.e., stand type) did not influence occupancy by reproductive females. It is unclear if silvicultural practices of partial harvest and patch retention would provide adequate hiding and escape cover for females with calves.

Stand age was not a predictor of general moose occupancy, but it was the strongest predictor of occupancy by females with calves. We expected that moose of any sex or reproductive status would prefer regenerating stands (11–25 years) over newly logged stands (0–10 years) because of greater access to food and cover. Regenerating stands in our study area had more lateral cover and deciduous forage than newly logged stands (Figure A3). However, only females with calves showed evidence of a response to stand age. Mean model‐predicted occupancy by females with calves was approximately three times higher in regenerating and unsalvaged stands than in newly logged stands. However, the regression coefficient for stand age was not statistically significant, perhaps due to nonlinear effects of stand age categories. Mumma et al. (2021) found that female moose in British Columbia selected regenerating cutblocks (9–24 years) and avoided new cutblocks (0–8 years). Regenerating cutblocks are associated with high predation mortality risk, although moose may seek forage in cutblocks, likely creating an ecological trap (Boucher et al. 2022). Similarly, caribou in eastern Canada selected regenerating cutblocks but suffered high calf mortality from black bear predation in those habitats, which were also selected by bears (Dussault et al. 2012; Leblond et al. 2016). Use of regenerating cutblocks by reproductive female moose may come at a predation cost. Bears are responsible for 58% of moose calf mortality in southwest Yukon (Larsen et al. 1989), but we found no evidence that reproductive females avoided habitat with more frequent bear detections.

Linear disturbances improve access for moose predators and hunters (Rempel et al. 1997; Hebblewhite et al. 2009; Whittington et al. 2011) and may be associated with high perceived risk. Yet, linear disturbance density (predominantly roads) had no influence on moose occupancy in our study area, regardless of moose reproductive status. In contrast, others have found that roads alter the risk–reward landscape and contribute to sexually divergent habitat selection by moose (Laurian et al. 2012; Francis et al. 2021; Mumma et al. 2021). Yukon has a small development footprint and lower road densities than many harvested landscapes in the southern boreal forest (Poley et al. 2022). Nevertheless, road densities were above the reported threshold of 0.2 km/km^2^ when moose respond negatively (Beyer et al. 2013). Road avoidance may be a successful strategy to reduce encounters with wolves (Boucher et al. 2022) and human hunters, but not bears (Leblond et al. 2016). Perhaps road avoidance was not an antipredator strategy used by moose in our study area.

### 4.1 Methods Considerations

Our study design and analytical methods had limitations. We constrained our sampling to the snow‐free period from May to October, but this is when the vast majority of moose calf mortalities occur (Larsen et al. 1989). We did not model different seasons separately within this period (e.g., calving, summer, and fall) due to low moose detection rates, although we tested for monthly variation in moose detection probability and found none. Seasonal changes in moose habitat use can be significant in some regions (McCulley et al. 2017; Francis et al. 2021). Sexual segregation between reproductive and solitary moose may diminish throughout the summer as females lose calves (Miquelle et al. 1992). Female and male moose come together during the rut, from approximately mid‐September to mid‐October (Oehlers et al. 2011). Our multistate models compared occupancy of reproductive and solitary female moose, so conclusions about sexually divergent habitat preferences were not confounded by rut behavior. Additionally, we sought to examine fine‐scale patterns of moose habitat use in response to primarily stand‐level forest attributes. However, moose habitat preferences are scale‐dependent (Herfindal et al. 2009) and a study with a focus on broader, landscape‐scale occupancy patterns may yield different results.

Camera traps and multistate occupancy models were useful for detecting divergent habitat use by reproductive and solitary female moose, with some caveats. Had we neglected to examine state‐dependent habitat use by moose, our results would show only positive responses to canopy removal via salvage logging. The potential avoidance of newly logged stands by females with calves would have been obscured. However, our results were not as precise as similar studies done with GPS collars (e.g., Mumma et al. 2021). Multistate occupancy models are highly parameterized, owing to additional parameters estimated for state‐dependency and state uncertainty (Nichols et al. 2007). Parameter uncertainty can result from low statistical power (Bassing et al. 2023). In our study, low model precision sometimes precluded statistical significance—despite moderate to strong effect sizes—and precision may have been improved with more camera sites and multiyear surveys (Mackenzie and Royle 2005). GPS telemetry provides fine‐scale location data for habitat selection models, but collaring animals can be cost‐prohibitive, invasive to wildlife, and prone to small sample sizes (i.e., few collared animals) that provide poor population‐level inference (Hebblewhite and Haydon 2010; Kays et al. 2015). Importantly, moose in our region are a cultural keystone species (Jung et al. 2018), and capturing, handling, and placing GPS collars on them is not acceptable to many local First Nation people. In a direct comparison of camera traps and occupancy models versus GPS collars and resource selection functions, there was near‐universal agreement in the direction of effects, but camera results had smaller effect sizes and more parameter uncertainty (Bassing et al. 2023). Camera trapping is a suitable alternative for detecting state‐dependent habitat use—especially where a social license for capturing and GPS collaring is lacking—if adequate statistical power can be achieved.

## 5 Conclusions

Climate change is predicted to increase the frequency and severity of natural disturbances such as wildfires and bark beetle outbreaks (Berg et al. 2006; Bentz et al. 2010), generating more opportunities to salvage log. Salvage logging has potential positive and negative effects on moose distribution, depending on sex and reproductive status. Regenerating logged stands (> 10 years) may provide attractive forage and adequate cover. However, newly logged stands need adequate time to recover and regain their value to reproductive moose. Recovery timelines are highly dependent on ecosystem productivity (Gagné et al. 2016; Mumma et al. 2021). Our results suggest the importance of unlogged forest stands to females with calves, and calf survival is key to moose population growth. Landscape management to achieve mixed forest composition (disturbance type and regenerative stage) may best meet the food and cover requirements of moose, a conclusion that is strongly supported by the literature (reviewed in Johnson and Rea 2024). Salvage logging practices that support heterogeneous age structure and large patches of unlogged forest are more likely to support viable moose populations, although more research on fitness consequences of salvage logging is needed (e.g., Boucher et al. 2022).

## Author Contributions

**Julie P. Thomas:** conceptualization (equal), formal analysis (lead), funding acquisition (equal), investigation (lead), methodology (lead), writing – original draft (lead). **Mary L. Reid:** conceptualization (equal), funding acquisition (equal), methodology (supporting), supervision (equal), writing – review and editing (equal). **Robert M. R. Barclay:** conceptualization (equal), funding acquisition (equal), methodology (supporting), supervision (equal), writing – review and editing (equal). **Thomas S. Jung:** conceptualization (equal), funding acquisition (equal), methodology (supporting), resources (lead), supervision (equal), writing – review and editing (equal).

## Conflicts of Interest

The authors declare no conflicts of interest.

## Data Availability

The raw data that supports the findings of this study are available in the Dryad digital repository: https://doi.org/10.5061/dryad.bk3j9kdpn.

## Appendix A

**TABLE A1:** Full model selection results for single‐ and multi‐state moose occupancy (*ψ*).

Species/model	(Q)AIC	Δ(Q)AIC	(Q)AIC weight	*K*
Single‐state moose occupancy				
*ψ* (Canopy + CWD + Water distance), *ρ* (.)	244.56	0	0.66	5
*ψ* (Canopy + CWD), *ρ* (.)	248.14	3.58	0.11	4
*ψ* (Stand Type + CWD + Water distance), *ρ* (.)	249.46	4.9	0.057	5
*ψ* (Canopy + Water distance), *ρ* (.)	249.73	5.17	0.050	4
*ψ* (Stand Type + CWD), *ρ* (.)	250.67	6.11	0.031	4
*ψ* (Canopy), *ρ* (.)	250.99	6.43	0.026	3
*ψ* (Water distance + Stand type), *ρ* (.)	251.15	6.59	0.024	4
*ψ* (Stand age + CWD), *ρ* (.)	251.78	7.22	0.017	4
*ψ* (Stand type), *ρ* (.)	252.16	7.6	0.014	3
*ψ* (Deciduous stems), *ρ* (.)	252.68	8.12	0.011	3
*ψ* (Stand age), *ρ* (.)	253.2	8.64	0.008	3
*ψ* (Lateral cover + CWD), *ρ* (.)	253.59	9.03	0.007	4
*ψ* (.), *ρ* (.)	253.78	9.22	0.006	2
*ψ* (CWD), *ρ* (.)	254.75	10.19	0.004	3
*ψ* (Lateral cover), *ρ* (.)	254.97	10.41	0.003	3
*ψ* (Forest cover), *ρ* (.)	255.62	11.06	0.003	3
*ψ* (Linear density), *ρ* (.)	255.78	11.22	0.02	3
Multistate moose occupancy				
*ψ* ^1^ (Canopy + CWD), *ψ* ^2^ (Stand age), *ρ* (.), *δ* (.)	295.06	0	0.73	7
*ψ* ^1^ (Canopy + CWD), *ψ* ^2^ (Water distance + deciduous stems), *ρ* (.), *δ* (.)	300.03	4.97	0.061	8
*ψ* ^1^ (Canopy + CWD), *ψ* ^2^ (Deciduous stems), *ρ* (.), *δ* (.)	300.58	5.52	0.046	7
*ψ* ^1^ (Canopy + CWD), *ψ* ^2^ (Water distance + bears), *ρ* (.), *δ* (.)	300.62	5.56	0.045	8
*ψ* ^1^ (Canopy + CWD), *ψ* ^2^ (Water distance), *ρ* (.), *δ* (.)	301.14	6.08	0.035	7
*ψ* ^1^ (Canopy + CWD), *ψ* ^2^ (Stand type + bears), *ρ* (.), *δ* (.)	302.43	7.36	0.018	8
*ψ* ^1^ (Canopy + CWD), *ψ* ^2^ (Bears), *ρ* (.), *δ* (.)	302.97	7.91	0.014	7
*ψ* ^1^ (Canopy + CWD), *ψ* ^2^ (Lateral cover), *ρ* (.), *δ* (.)	303.01	7.95	0.014	7
*ψ* ^1^ (Canopy + CWD), *ψ* ^2^ (Stand type), *ρ* (.), *δ* (.)	303.01	7.95	0.014	7
*ψ* ^1^ (Canopy + CWD), *ψ* ^2^ (Forest cover), *ρ* (.), *δ* (.)	303.02	7.96	0.014	7
*ψ* ^1^ (Canopy + CWD), *ψ* ^2^ (Linear density), *ρ* (.), *δ* (.)	303.07	8.01	0.013	7
*ψ* ^1^ (.), *ψ* ^2^ (.), *ρ* (.), *δ* (.)	310.72	15.65	0.000	4

*Note:* Quasi‐Akaike's Information Criterion (QAIC), delta QAIC, QAIC weight, and number of parameters (*K*) are shown. Multistate models include two occupancy states: (*ψ* ^1^) Probability of occupancy by adult females, and (*ψ* ^2^) probability of occupancy by females with calves. *ρ* = Probability of detection of adult females (True state = 1 or 2), and *δ* = Probability that calves are detected, given occupancy (True state =2). Predictors are defined in Table 1 and (.) refers to the null model.

**TABLE A2:** Model averaged parameter estimates, unconditional standard errors (SE), and 95% confidence intervals (CI) for general moose detection (*ρ*) and single‐state occupancy (*ψ*) in beetle‐affected and salvage‐logged forest in Yukon, Canada.

Species/parameter	Estimate	SE	95% lower CI	95% upper CI
*ρ* intercept	−1.09	0.48	−2.03	−0.15
*Ψ* intercept	1.83	1.39	−0.9	4.56
**Canopy**	−1.85	0.94	−3.69	−0.01
Water distance	−1.49	0.86	−3.18	0.20
CWD	−1.84	1.02	−3.85	0.16
Stand type	−1.82	1.18	−4.12	0.49

*Note:* Predictor variables following *ρ* and *ψ* intercepts are detection and occupancy parameters, respectively. Estimates were calculated by averaging across the set of confidence models where QAIC or AIC weights summed to 0.95. Occupancy predictors are defined in Table 1. Significant coefficient estimates (CI'S do not overlap zero) are listed in bold.

**TABLE A3:** Model averaged parameter estimates, unconditional standard errors (SE), and 95% confidence intervals (CI) for moose multistate models.

Parameter	Estimate	SE	95% lower CI	95% upper CI
Probability of occupancy (*ψ* ^1^)				
ψ1 intercept	0.94	0.95	−0.92	2.80
**Canopy**	−1.71	0.77	−3.21	−0.21
CWD	−1.68	0.93	−3.49	0.13
Probability of calving, given occupancy (*ψ* ^2^)				
*ψ*2 intercept	−1.21	1.80	−4.74	2.32
Stand Age	3.76	2.67	−1.45	8.97
Deciduous Stems	−2.01	1.41	−4.77	0.76
Water distance	1.96	1.36	−0.70	4.62
Bears	1.47	1.68	−1.84	4.77
Stand type	1.60	1.07	−0.49	3.69
Probability of detection (*ρ*, *δ*)				
*ρ* intercept	−1.52	0.25	−2.01	−1.03
*δ* intercept	−0.15	0.55	−1.23	0.93

*Note:* Models include two occupancy states: (*ψ* ^1^) Probability of female occupancy, and (*ψ* ^2^) Probability of calves, given that a site is occupied by females. *ρ* = Probability of detection of adult females (True state = 1 or 2), and *δ* = Probability that calves are detected, given occupancy (True state = 2). Significant coefficient estimates (Confidence intervals do not overlap zero) are in bold.
